# Supplementary material for: Multistability and anomalies in oscillator models of lossy power grids
Source: Nat Commun. 2022 Sep 6;13:5238. doi: 10.1038/s41467-022-32931-8 (PMC9448753; doi:10.1038/s41467-022-32931-8)
Supplement: Supplementary file 1 — Supplementary Information [file 41467_2022_32931_MOESM1_ESM.pdf]

Supplementary Information for
Multistability and Anomalies in Oscillator Models of Lossy Power Grids

Robin Delabays,* Saber Jafarpour, and Francesco Bullo

*Corresponding author. Email: robindelabays@ucsb.edu

Content

Section S1: The proof of Theorem 3;

Section S2: The proof of Theorem 5;

Section S3: Additional bounds on the algebraic connectivity.

Section S1. Proof of Theorem 3

Without loss of generality, let us renumber the nodes such that node 1 is a leaf of the graph G_u, and such that, for all $i \in \{2, ..., n-1\}$, node i is a leaf of the subgraph $G_i \subset G_\mathrm{u}$ where nodes with index up to $i-1$ are pruned. Furthermore, let us denote by e_i the unique edge that connects node $i \in \{1, ..., n-1\}$ to the set of nodes $\{i+1, ..., n\}$, and orient it such that i is the source of e, i.e., $s_{e_i} = i$. The reversed edge is denoted $e_{i+m} = \bar{e}_i$. All directed edges are then indexed. This can be done iteratively and we provide an implementation of this renumbering in Ref. 1.

For convenience, we recall and slightly rephrase equation (15a),

$$\varphi = \omega_j - \sum_{\substack{e:\\ s_e=j}} h_e(\Delta_e)\,, \qquad j \in \{1, ..., n\},\ \varphi \in \mathbb{R}\,, \tag{S1a}$$

$$|\Delta_e| \leq \gamma_e\,, \qquad e \in E_\mathrm{u}\,. \tag{S1b}$$

A solution $\mathbf{\Delta}$ of equation (15a) is also a solution of equations (S1). Showing that there is at most one solution to equations (S1) then implies that there is at most one solution to equation (15a).

Assume that $\mathbf{\Delta}$ and $\mathbf{\Delta}'$ are two solutions of equation (15a), with respective synchronous frequencies φ and φ'. Without loss of generality, assume that $\varphi \leq \varphi'$. We compare now the components of the two solutions recursively.

Initial step, $i = 1$. According to our choice of indexing, equation (S1a) gives

$$h_{e_1}(\Delta_{e_1}) = \omega_1 - \varphi \geq \omega_1 - \varphi' = h_{e_1}(\Delta'_{e_1})\,, \tag{S2}$$

and by monotonicity of the coupling functions,

$$\Delta_{e_1} \geq \Delta'_{e_1}\,. \tag{S3}$$

Recursion step, $2 \leq i \leq n-1$. By the previous steps, we have $\Delta_{e_j} \geq \Delta'_{e_j}$ for $j \in \{1, ..., i-1\}$. According to our choice of indexing, there is a single out-going edge from node i whose angle differences in the two solutions have not been compared in the previous steps, namely e_i (see Fig. S1). Again by equation (S1a) and monotonicity of the coupling functions, we get

$$h_{e_i}(\Delta_{e_i}) = \omega_i - \sum_{\substack{e:s_e<i,\\ t_e=i}} h_{\bar{e}}(-\Delta_e) - \varphi \geq \omega_i - \sum_{\substack{e:s_e<i,\\ t_e=i}} h_{\bar{e}}(-\Delta'_e) - \varphi' = h_{e_i}(\Delta'_{e_i})\,, \tag{S4}$$

and

$$\Delta_{e_i} \geq \Delta'_{e_i}\,. \tag{S5}$$

Final step, $i = n$. All the previous steps together with equation (S1a) and monotonicity of the coupling functions give

$$\varphi = \omega_n - \sum_{\substack{e:\\ t_e=n}} h_{\bar{e}}(-\Delta_e) \geq \omega_n - \sum_{\substack{e:\\ t_e=n}} h_{\bar{e}}(-\Delta'_e) = \varphi' \geq \varphi\,, \tag{S6}$$

which implies $\varphi = \varphi'$. The inequalities in equations (S2), (S3), (S4), (S5), and (S6) are then equalities and the two solutions are identical. Note that we crucially used that the coupling functions are strictly increasing.

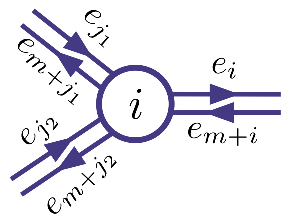

FIG. S1. **Illustration of the node and edge indexing.** In this example, our construction implies $j_1, j_2 < i \leq m$.

Section S2. Proof of Theorem 5

For technical purposes, we need to define the extended flow function $\mathbf{h}_\gamma\colon \mathbb{R}^m \to \mathbb{R}^{2m}$, whose components are straight lines extensions of the components of $\mathbf{h}$. For each edge e,

$$[\mathbf{h}_\gamma(\mathbf{y})]_e = \begin{cases} h_e'(-\gamma_e)(y_e+\gamma_e) + h_e(-\gamma_e)\,, & y_e < -\gamma_e\,,\\ h_e(y_e)\,, & |y_e| \leq \gamma_e\,,\\ h_e'(\gamma_e)(y_e-\gamma_e) + h_e(\gamma_e)\,, & y_e > \gamma_e\,,\end{cases} \tag{S7}$$

$$[\mathbf{h}_\gamma(\mathbf{y})]_{e+m} = \begin{cases} -h_{\bar e}'(\gamma_e)(y_e+\gamma_e) + h_{\bar e}(\gamma_e)\,, & y_e < -\gamma_e\,,\\ h_{\bar e}(-y_e)\,, & |y_e| \leq \gamma_e\,,\\ -h_{\bar e}'(-\gamma_e)(y_e-\gamma_e) + h_{\bar e}(-\gamma_e)\,, & y_e > \gamma_e\,,\end{cases} \tag{S8}$$

which is well-defined and continuously differentiable.

Let $\boldsymbol{\xi}, \boldsymbol{\eta} \in \mathbb{R}^m$, such that $C_\Sigma \boldsymbol{\xi} = C_\Sigma \boldsymbol{\eta} = \mathbf{u} \in \mathbb{Z}^c$, and define $\mathbf{y} = \boldsymbol{\xi} - \boldsymbol{\eta} \in \mathrm{Ker}(C_\Sigma)$. We construct the two diagonal $m \times m$ matrices $\Lambda_1(\boldsymbol{\xi},\boldsymbol{\eta})$, $\Lambda_2(\boldsymbol{\xi},\boldsymbol{\eta})$ as

$$(\Lambda_1)_e = (\xi_e - \eta_e)^{-1} \int_{\eta_e}^{\xi_e} (\mathbf{h}_\gamma)_e'(t)dt\,, \tag{S9}$$

$$(\Lambda_2)_e = (\xi_e - \eta_e)^{-1} \int_{\eta_e}^{\xi_e} (\mathbf{h}_\gamma)_{\bar e}'(-t)dt\,. \tag{S10}$$

We verify that

$$\mathbf{h}_\gamma(\boldsymbol{\xi}) - \mathbf{h}_\gamma(\boldsymbol{\eta}) = \begin{pmatrix} \Lambda_1 \\ -\Lambda_2 \end{pmatrix} (\boldsymbol{\xi} - \boldsymbol{\eta}) = \Lambda \mathbf{y}\,, \tag{S11}$$

and the two matrices Λ_1, Λ_2 are nonnegative, because the coupling functions are assumed strictly increasing.

Then, by definition of S_ϵ,

$$\|S_\epsilon(\xi) - S_\epsilon(\eta)\|_2^2 = \mathbf{y}^\top \left(I_m - \epsilon B_\mathrm{u}^\top L_\mathrm{u}^\dagger B_\mathrm{o} \Lambda\right)^\top \left(I_m - \epsilon B_\mathrm{u}^\top L_\mathrm{u}^\dagger B_\mathrm{o} \Lambda\right) \mathbf{y} = \|\mathbf{y}\|_2^2 - \epsilon \mathbf{y}^\top M \mathbf{y} + O(\epsilon^2)\,, \tag{S12}$$

where

$$M = B_\mathrm{u}^\top L_\mathrm{u}^\dagger B_\mathrm{o} \Lambda + \Lambda^\top B_\mathrm{o}^\top (L_\mathrm{u}^\dagger)^\top B_\mathrm{u}\,. \tag{S13}$$

The remainder of the proof will show that, under the assumptions of the theorem, $\mathbf{y}^\top M \mathbf{y} > 0$. Then for $\epsilon > 0$ small enough, the second order term in equation (S12) is dominated by the first order term, which is negative. Therefore, equation (S12) is strictly smaller than $\|\mathbf{y}\|_2^2$, and S_ϵ is contracting.

We remark that $B_\mathrm{o}\Lambda$ has exactly the same sparsity and sign pattern as B_u, with the difference being that the nonzero terms are not all the same, not even for the terms corresponding to the ends of a given edge. Namely,

$$(B_\mathrm{o}\Lambda)_{ie} = \begin{cases} (\Lambda_1)_{ee}\,, & \text{if } e = (i,j) \text{ for some } j\,,\\ -(\Lambda_2)_{ee}\,, & \text{if } e = (j,i) \text{ for some } j\,,\\ 0\,, & \text{otherwise.}\end{cases} \tag{S14}$$

We illustrate this property for a simple network in the example below.

We now define

$$\Lambda_\mathrm{p} = \frac{\Lambda_1 + \Lambda_2}{2}\,, \qquad \Lambda_\mathrm{m} = \frac{\Lambda_1 - \Lambda_2}{2}\,, \tag{S15}$$

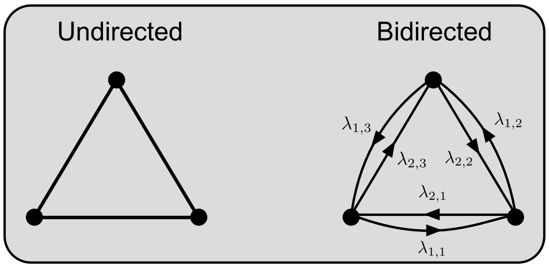

FIG. S2. **Triangle network.** Illustration of the example's triangle network.

and recall that the out-incidence matrix is given by $B_\mathrm{o} = ([B_\mathrm{u}]_+, [B_\mathrm{u}]_-)$ in equation (25), where the square brackets denote the positive and negative parts (see the Methods Section for details). Then we can rewrite

$$B_\mathrm{o}\Lambda = [B_\mathrm{u}]_+\Lambda_1 - [B_\mathrm{u}]_-\Lambda_2 = [B_\mathrm{u}]_+(\Lambda_\mathrm{p} + \Lambda_\mathrm{m}) - [B_\mathrm{u}]_-(\Lambda_\mathrm{p} - \Lambda_\mathrm{m}) = B_\mathrm{u}\Lambda_\mathrm{p} + |B_\mathrm{u}|\Lambda_\mathrm{m}\,, \tag{S16}$$

with the absolute value taken elementwise.

Example. We illustrate equations (S14) and (S16) for the case of a triangular network, shown in Fig. S2. The matrices involved are

$$B_\mathrm{o} = \begin{pmatrix} 1 & 0 & 0 & 0 & 0 & 1 \\ 0 & 1 & 0 & 1 & 0 & 0 \\ 0 & 0 & 1 & 0 & 1 & 0 \end{pmatrix}, \qquad \Lambda = \begin{pmatrix} \lambda_{1,1} & 0 & 0 \\ 0 & \lambda_{1,2} & 0 \\ 0 & 0 & \lambda_{1,3} \\ -\lambda_{2,1} & 0 & 0 \\ 0 & -\lambda_{2,2} & 0 \\ 0 & 0 & -\lambda_{2,3} \end{pmatrix}, \qquad B_\mathrm{o}\Lambda = \begin{pmatrix} \lambda_{1,1} & 0 & -\lambda_{2,3} \\ -\lambda_{2,1} & \lambda_{1,2} & 0 \\ 0 & -\lambda_{2,2} & \lambda_{1,3} \end{pmatrix}, \tag{S17}$$

illustrating equation (S14). Now defining $\lambda_{\mathrm{p},i} = (\lambda_{1,i} + \lambda_{2,i})/2$ and $\lambda_{\mathrm{m},i} = (\lambda_{1,i} - \lambda_{2,i})/2$, we can decompose $B_\mathrm{o}\Lambda$ as

$$B_\mathrm{o}\Lambda = \begin{pmatrix} \lambda_{\mathrm{p},1} & 0 & -\lambda_{\mathrm{p},3} \\ -\lambda_{\mathrm{p},1} & \lambda_{\mathrm{p},2} & 0 \\ 0 & -\lambda_{\mathrm{p},2} & \lambda_{\mathrm{p},3} \end{pmatrix} + \begin{pmatrix} \lambda_{\mathrm{m},1} & 0 & \lambda_{\mathrm{m},3} \\ \lambda_{\mathrm{m},1} & \lambda_{\mathrm{m},2} & 0 \\ 0 & \lambda_{\mathrm{m},2} & \lambda_{\mathrm{m},3} \end{pmatrix},$$

which is precisely equation (S16).

Based on equations (S13) and (S16), on the fact that $\mathbf{y} = B_\mathrm{u}^\top L^\dagger B_\mathrm{u}\mathbf{y}$ (see Prop. S1), and using $\mathbf{z} = L_\mathrm{u}^\dagger B_\mathrm{u}\mathbf{y}$, we compute

$$\mathbf{y}^\top M\mathbf{y} = \mathbf{z}^\top \left(B_\mathrm{o}\Lambda B_\mathrm{u}^\top + B_\mathrm{u}\Lambda B_\mathrm{o}^\top\right)\mathbf{z} = \mathbf{z}^\top \left(2B_\mathrm{u}\Lambda_\mathrm{p}B_\mathrm{u}^\top + |B_\mathrm{u}|\Lambda_\mathrm{m}B_\mathrm{u}^\top + B_\mathrm{u}\Lambda_\mathrm{m}|B_\mathrm{u}|^\top\right)\mathbf{z} = 2\mathbf{z}^\top\left(L_\mathrm{p} + D_\mathrm{m}\right)\mathbf{z}\,, \tag{S18}$$

where

$$L_\mathrm{p} = B_\mathrm{u}\Lambda_\mathrm{p}B_\mathrm{u}^\top \tag{S19}$$

$$D_\mathrm{m} = \left(|B_\mathrm{u}|\Lambda_\mathrm{m}B_\mathrm{u}^\top + B_\mathrm{u}\Lambda_\mathrm{m}|B_\mathrm{u}|^\top\right)/2\,. \tag{S20}$$

First, notice that L_p is a weighted Laplacian matrix with the graph structure of G_u. The weight of edge e is given by

$$(\Lambda_\mathrm{p})_{ee} = (\xi_e - \eta_e)^{-1}\int_{\eta_e}^{\xi_e} f_e'(t)dt\,, \tag{S21}$$

which, by the Mean Value Theorem, is bounded by

$$\inf_x f_e'(x) \leq (\Lambda_\mathrm{p})_e \leq \sup_x f_e'(x)\,, \tag{S22}$$

with the infimum and supremum taken over the admissible values of x. Therefore, by definition of the odd weighted Laplacian matrix [equation (41)], we can bound the second eigenvalue of L_p by Weyl's inequalities,[2]

$$\lambda_2(L_\mathrm{p}) \geq \inf_\mathbf{x} \lambda_2\left(L_f(\mathbf{x})\right)\,. \tag{S23}$$

Finally, using that $\mathbf{z} = L^{\dagger} B_{\mathrm{u}} \mathbf{y}$ is orthogonal to the null space of L_{p},

$$\mathbf{z}^\top L_{\mathrm{p}} \mathbf{z} \geq \lambda_2(L_{\mathrm{p}}) \|\mathbf{z}\|_2^2 \geq \inf_{\mathbf{x}} \lambda_2(L_f(\mathbf{x})) \|\mathbf{z}\|_2^2 \,. \tag{S24}$$

Second, let us break down the matrix D_{m}. Using the positive and negative parts of the incidence matrix, $B_{\mathrm{u}} = [B_{\mathrm{u}}]_+ - [B_{\mathrm{u}}]_-$ and $|B_{\mathrm{u}}| = [B_{\mathrm{u}}]_+ + [B_{\mathrm{u}}]_-$, direct computation shows

$$D_{\mathrm{m}} = [B_{\mathrm{u}}]_+ \Lambda_{\mathrm{m}} [B_{\mathrm{u}}]_+^\top - [B_{\mathrm{u}}]_- \Lambda_{\mathrm{m}} [B_{\mathrm{u}}]_-^\top \,. \tag{S25}$$

According to Prop. 1, we know that D_{m} is diagonal, and its elements can be computed as

$$(D_{\mathrm{m}})_{ii} = \sum_e \left([B_{\mathrm{u}}]_+\right)_{ie}^2 (\Lambda_{\mathrm{m}})_{ee} - \sum_e \left([B_{\mathrm{u}}]_-\right)_{ie}^2 (\Lambda_{\mathrm{m}})_{ee} = \sum_e \left([B_{\mathrm{u}}]_+ - [B_{\mathrm{u}}]_-\right)_{ie} (\Lambda_{\mathrm{m}})_{ee} = \sum_{e \in E_i} \pm (\Lambda_{\mathrm{m}})_{ee} \,, \tag{S26}$$

where E_i is the set of edges incident to node i in G_{u}. Note that the elements of Λ_{m} are given by

$$(\Lambda_{\mathrm{m}})_e = (\xi_e - \eta_e)^{-1} \int_{\eta_e}^{\xi_e} g_e'(t) dt \,, \tag{S27}$$

and, by the Mean Value Theorem, are bounded by

$$|(\Lambda_{\mathrm{m}})_e| \leq \sup_x |g_e'(x)| \,. \tag{S28}$$

Therefore, by definition of the even weighted degree matrix [equation (42)], we can bound the elements of D_{m},

$$\min_i (D_{\mathrm{m}})_{ii} \geq -\sup_{\mathbf{x},i} (D_g(\mathbf{x}))_{ii} \,, \tag{S29}$$

which gives the bound

$$\mathbf{z}^\top D_{\mathrm{m}} \mathbf{z} \geq \min_i \, (D_{\mathrm{m}})_{ii} \, \|\mathbf{z}\|_2^2 \geq -\sup_{\mathbf{x},i} \, (D_g(\mathbf{x}))_{ii} \, \|\mathbf{z}\|_2^2 \,. \tag{S30}$$

To conclude, we introduce equations (S24) and (S30) into equation (S18), yielding

$$\mathbf{y}^\top M \mathbf{y} \geq \left[\inf_{\mathbf{x}} \lambda_2 \left(L_f(\mathbf{x})\right) - \sup_{\mathbf{x},i} \left(D_g(\mathbf{x})\right) \right] \|\mathbf{z}\|_2^2 \,, \tag{S31}$$

which is strictly positive under the assumptions of the theorem. Going back to equation (S12), we have shown that the first order term is strictly negative, and therefore, for $\epsilon > 0$ sufficiently small, the flow mismatch iteration S_ϵ is contracting, which concludes the proof.

Proposition S1. Let G be a graph and define its incidence matrix B, Laplacian matrix L, and the cycle-edge incidence matrix C_Σ (see the Methods Section for definitions). Then

$$\mathrm{Ker}(C_\Sigma) = \mathrm{Ker}(I_m - B^\top L^\dagger B) \,. \tag{S32}$$

Proof. Let $\mathbf{x} \in \mathrm{Ker}(I_m - B^\top L^\dagger B)$, then $\mathbf{x} = B^\top L^\dagger B \mathbf{x}$. We compute

$$C_\Sigma \mathbf{x} = C_\Sigma B^\top L^\dagger B \mathbf{x} = 0 \,, \tag{S33}$$

because $C_\Sigma B^\top = 0$. Thus $\mathrm{Ker}(I_m - B^\top L^\dagger B) \subset \mathrm{Ker}(C_\Sigma)$.

The rows of C_Σ are linearly independent by definition. Then its kernel has dimension $m - (m - n + 1) = n - 1$. The matrix $I_m - B^\top L^\dagger B$ is the orthogonal projection onto the kernel of B, therefore its rank is the nullity of B. By the Rank-Nullity Theorem,

$$\mathrm{null}(I_m - B^\top L^\dagger B) = m - \mathrm{null}(B) = n - 1 \,. \tag{S34}$$

The two kernel have the same dimension.

The set $\mathrm{Ker}(I_m - B^\top L^\dagger B)$ is then a subspace of $\mathrm{Ker}(C_\Sigma)$ and has the same dimension, they are then identical.

Section S3. Bounds on the algebraic connectivity

The following bounds are adapted from standard results of algebraic graph theory. We summarize the bounds of interest and the ad hoc quantities in Table S1.

Proposition S2. With the definition of L_f given in equation (41), we have the following bounds on its Fiedler eigenvalue:

(i) $\lambda_2 \geq 2c_\mathrm{e}[1-\cos(\pi/n)]$ (Ref. 3, paragraph 4.3);

(ii) $\lambda_2 \geq (nd_\mathrm{w})^{-1}$ (adapted from Ref. 4, Lemma 1.9);

(iii) $\lambda_2 \geq 4\min_e \sigma_{\mathrm{min},e}/nd_{G_\mathrm{u}}$ (adapted from Ref. 5, Theorem 4.2).

All relevant quantities are defined in Tab. S1.

Proof. (i). Defining the weighted edge connectivity,[3]

$$c(\mathbf{x}) = \min_{\mathcal{S}} \sum_e f'_e(x_e)\,, \tag{S35}$$

where the minimum is taken over subsets of edges $\mathcal{S} \subset E_\mathrm{u}$ that split the graph G_u, we can adapt the proof of Ref. 3, in paragraph 4.3, yielding

$$\lambda_2(L_f) \geq 2c(\mathbf{x})\,[1-\cos(\pi/n)] \geq 2c_\mathrm{e}\,[1-\cos(\pi/n)]\ , \tag{S36}$$

independently of $\mathbf{x}$, where c_e is defined in Tab. S1.

(ii). We adapt here the proof of Ref. 4, Lemma 1.9. Let $\mathbf{v}$ be the eigenvector of L_f associated with λ_2 and assume that $|v_i| = \max_k |v_k|$ (recall that all these quantities depend on $\mathbf{x}$). Because L_f is a symmetric Laplacian matrix, $\mathbf{1}^\top \mathbf{v} = 0$ and there is an index j such that $v_i v_j < 0$. We denote with P_{ij} the shortest (weighted) path from i to j. Now,

$$\lambda_2 = \frac{\mathbf{v}^\top L_f \mathbf{v}}{\mathbf{v}^\top \mathbf{v}} = \frac{\sum_{e=(k,\ell)} f'_e(x_e)(v_k - v_\ell)^2}{\sum_k v_k^2} \geq \sum_{e=(k,\ell)\in P_{ij}} \frac{f'_e(x_e)(v_k-v_\ell)^2}{n v_i^2}\,. \tag{S37}$$

Defining the odd weighted diameter

$$D_\mathrm{w}(\mathbf{x}) = \max_{i,j} \min_{P_{ij}} \sum_{e\in P_{ij}} [f'_e(x_e)]^{-1}\,, \tag{S38}$$

where the maximum is taken over all pairs of vertices and the minimum is taken over all simple paths joining i and j, we can apply the Sedrakyan inequality[6] (direct consequence of the Cauchy-Schwarz inequality) to the numerator of equation (S37) and get

$$\lambda_2 \geq \frac{[D_\mathrm{w}(\mathbf{x})]^{-1}(v_i - v_j)^2}{n v_i^2} \geq \frac{1}{nD_\mathrm{w}(\mathbf{x})} \geq \frac{1}{nd_\mathrm{w}}\,, \tag{S39}$$

where d_w is defined in Tab. S1.

(iii). Alternatively, both sides of the identity

$$\mathbf{v}^\top L_f \mathbf{v} = \lambda_2 \mathbf{v}^\top \mathbf{v}\,, \tag{S40}$$

can be bounded as follows,

$$\mathbf{v}^\top L_f \mathbf{v} = \sum_{e=(i,j)\in E_\mathrm{u}} f'_e(x_e)(v_i - v_j)^2 \geq \min_e \sigma_{\mathrm{min},e}\,, \tag{S41}$$

and

$$\begin{aligned} 2n\mathbf{v}^\top \mathbf{v} &= \sum_i \sum_j (v_i - v_j)^2 \leq \sum_i \sum_j |P_{ij}| \sum_{(k,\ell)\in P_{ij}} (v_k - v_\ell)^2 \leq \sum_{(k,\ell)\in E_\mathrm{u}} (v_k - v_\ell)^2 d_{G_\mathrm{u}} \sum_i \sum_j \chi_{ij}(k,\ell) \\ &\leq \sum_{(k,\ell)\in E_\mathrm{u}} (v_k - v_\ell)^2 d_{G_\mathrm{u}} n^2/2\,, \end{aligned} \tag{S42}$$

Bounds	Reference
$\max_i (D_g)_{ii} \leq \max_i \sum_{e\in E_i} \alpha_{\max,e}$	Direct computation (E_i is the set of edges incident to node i).
$\lambda_2 \geq 2c_{\rm e}[1-\cos(\pi/n)]$	Prop. S2, adapted from Ref. 3, paragraph 4.3).
$\lambda_2 \geq (nd_{\rm w})^{-1}$	Prop. S2, adapted from Lemma 1.9 in Ref. 4.
$\lambda_2 \geq 4\min_e \sigma_{\min,e}/nd_{G_{\rm u}}$	Prop. S2, adapted from Theorem 4.2 in Ref. 5.

Definitions	Name
$\alpha_{\max,e} = \sup_x \lvert g_e'(x)\rvert$	Maximal even slope.
$\sigma_{\min,e} = \inf_x f_e'(x)$	Minimal odd slope.
$c_{\rm e} = \min_{E\subset\mathcal{S}} \sum_{e\in E} \sigma_{\min,e}$	Minimized odd weighted edge connectivity ($\mathcal{S}$ is the set of splitting edge sets).
$d_{\rm w} = \max_{i,j} \min_{P_{ij}} \sum_{e\in P_{ij}} \sigma_{\min,e}^{-1}$	Maximized odd weighted diameter (P_{ij} denotes a path between i and j).
$d_{G_{\rm u}} = \max_{i,j} \min_{P_{ij}} \lvert P_{ij}\rvert$	Graph diameter (P_{ij} denotes a path between i and j).

TABLE S1. List of bounds on the components of equation (18) and definition of the ad hoc quantities. Note that all quantities are independent of the state of the system and can be determined beforehand.

where we used that $\mathbf{v}^\top\mathbf{1} = 0$ and the Cauchy-Schwartz inequality at the first line, P_{ij} is a chosen (unweighted) shortest path between i and j, $\chi_{ij}(e)$ is its indicator function, and we used Lemma 4.1 in Ref. 5 at the last inequality. Plugging these bounds together yields

$$2n\min_e \sigma_{\min,e} \leq \lambda_2 d_{G_{\rm u}} n^2/2\,, \tag{S43}$$

which concludes the proof.

Supplementary References

1. R. Delabays, "DFNSolver: Dissipative Flow Networks Solver (v1.1), Zenodo," (2022).
2. R. A. Horn and C. R. Johnson, *Matrix Analysis* (Cambridge University Press, New York, 1994).
3. M. Fiedler, "Algebraic connectivity of graphs," Czech. Math. J. **23**, 298–305 (1973).
4. F. R. K. Chung, *Spectral Graph Theory*, CBMS Regional Conference Series in Mathematics ; Number 92 (Published for the Conference Board of the Mathematical Sciences by the American Mathematical Society, Providence, Rhode Island, 1997).
5. B. Mohar, "Eigenvalues, diameter, and mean distance in graphs," Graphs and Combin. **7**, 53–64 (1991).
6. H. Sedrakyan and N. Sedrakyan, *Algebraic Inequalities*, Problem Books in Mathematics (Springer International Publishing, Cham, 2018).
